# Quality of Life, Sexual Health, and Associated Factors Among the Sexually Active Adults in a Metro City of India: An Inquiry During the COVID-19 Pandemic-Related Lockdown

**DOI:** 10.3389/fpsyt.2022.791001

**Published:** 2022-03-24

**Authors:** Seshadri Sekhar Chatterjee, Ranjan Bhattacharyya, Amrita Chakraborty, Arista Lahiri, Abhijit Dasgupta

**Affiliations:** ^1^Department of Psychiatry, Diamond Harbour Government Medical College and Hospital, Diamond Harbour, India; ^2^Department of Psychiatry, Murshidabad Medical College and Hospital, Berhampore, India; ^3^Dr. B. C. Roy Multi-Speciality Medical Research Centre, Indian Institute of Technology Kharagpur, Kharagpur, India; ^4^Department of Medicine, University College Dublin, Dublin, Ireland

**Keywords:** sexual dysfunction, anxiety, depression, stress, psychosexual, survey, pandemic, quality of life

## Abstract

**Background:**

Sexual dysfunction (SD) and its effect on our life is an important but less studied topic especially during post-COVID era. This study examines the extent of SD and other mental health predictors and their effect on quality of life.

**Methods:**

A cross-sectional survey of sexually active adults was conducted in an Indian metro-city. Along with sociodemographic data, sexual dysfunction, depression, anxiety, stress, and quality of life were assessed by Arizona Sexual Experience Scale (ASEX), Depression Anxiety and Stress Scale (DASS), and WHOQOL-BREF, respectively. Structural equations modeling was used to understand their relationship.

**Results:**

Out of the total 1,376 respondents, 80.52% were male, 65.98% were married, and 48.54% were graduates. The mean age of the participants was 34.42 (±9.34) years. Of the participants, 27.18% had sexual dysfunction. Majority of the respondents did not have depression (59.30%), anxiety (52.33%), or stress (44.48%). Mild and moderate levels were the commonest findings among those who had depression, anxiety, or stress. Among the respondents, 27.18% had sexual dysfunction as per the ASEX instrument. Increase in age and female gender were associated with sexual dysfunction overall and also all its components. Presence of depression adversely affected ease of achieving orgasm and satisfaction from orgasm and was associated with sexual dysfunction overall. The respondents had a mean score of 73.57 (±13.50) as per the WHO-QOL. Depression and stress emerged as statistically significant factors for poor quality of life, while sexual dysfunction was not associated statistically.

**Conclusion:**

More than one-fourth of the study population reported sexual dysfunction during the first wave of the pandemic in India. The study findings highlight the role of poor mental health issues in this regard. In fact, issues like depression and stress were associated with poor quality of life as well. The current findings unequivocally warrant specific interventions to improve mental health of the respondents.

## Introduction

Sexual wellbeing is essential for maintaining overall physical and mental health through a variety of biological and psychological processes (1–3). Regular sexual activity reduces stress, regularizes sleep cycle, and regulates our mental wellbeing as a whole. On the contrary, the persistent depression and anxiety can affect sexual health (SH), which in turn lowers our quality of life (4). Following the coronavirus disease 2019 (COVID-19) spread, complete lockdown for a long time, new social norms, quarantine- and isolation-related issue, and even unpredictable geopolitical situations has affected the lives of the citizens (5). Several mental-health-related issues were identified in India during the lockdown period (6). Physical distancing, lack of peer group interaction, isolation during incubation days, “touch hunger,” and relationship problems all can directly or indirectly affect SH.

Mental health significantly influences sexual behavior, including sexual intercourse frequency. Generally, perceived wellbeing and mental health are positively associated with sexual pleasure and intercourse. Sometimes people with stress might get engaged in excessive sexual activity to reduce their stress levels. Alternatively, excessive worry and decreased motivation because of stress can also reduce sexual interest, arousal, and intercourse frequency (7). A scoping review on SH (excluding reproductive health, intimate partner violence, and gender-based violence) and COVID-19 by Kumar et al. finds very few studies done on the same subjects in low-/middle-income countries (LMICs) (8). SH has been neglected during any disaster, as other, more basic issues like primary medical care, safety, and nutrition become the priority (9). Any disasters will impair SH, which can be due to poor access to SH services and supplies, disrupted health facilities, reduced human resources, impoverishment, and exposure to sexual violence (10).

The past works reporting the investment in SH services following disasters are low leading to higher unmet needs (11). Although there are some studies already conducted on sexual health, only few empirical studies have focused on the associated factors. Studies done among various physiological and demographic groups during the pandemic have highlighted the role of depressive symptoms, anxiety, stress, and clinico-behaviors including addictions for being associated with SH (12–17). Often, sexual and mental health issues have been studied together (18). The existing literature, both during and before the pandemic, informs the importance of depression, anxiety, stress, different comorbidities, addiction habits like smoking, and social norms like marriage, etc. in the backdrop of age and gender, for understanding SH and the emergence or severity of sexual dysfunction (SD). The evidence in Indian context is even lesser, which brings forward the need for understanding the role of these factors, which are essentially modifiable in sexual dysfunction among the adult sexually active participants.

SD alone and also in synchrony with mental health conditions, physical ill health, and addictions impacts the overall quality of life of an individual (19–22). Researchers have explored the continuum of mental health, sexual dysfunction, and quality of life using path analysis as well (21). However, this continuum is not well explored amidst the COVID-19 pandemic. In addition, the role of practice of COVID-appropriate behaviors, economic loss, loss of job during lockdown, etc. appear to be plausible factors leading to a poor quality of life. Thus, not only the contributors of SD but also their role in synergy with SD, and different socio-behavioral factors' role on quality of life are important contemporary considerations. The most useful analytical technique in this regard to study this continuum and explore the effect sizes for different contributors, also taking into account the endogeneity of SD in the continuum path, is structural equations modeling (SEM) (23–25). In this backdrop, the present study was conducted to measure the proportion of sexual dysfunction among the study population and determine its association with different psychiatric morbidities. This article also explored the association of sexual dysfunction with quality of life of the participants.

## Methods

### Study Design and Participants

A cross-sectional study was carried out among the adult population aged 18 years and above residing in a Metropolitan city of Eastern India (Kolkata). Following approval from the Institutional Ethics Committee, an online questionnaire was distributed, with a digital consent form attached to it. Study population consisted of adults. Only those who provided consent could access the questionnaire. Those who ultimately completed the whole questionnaire were included. Eligible respondents but with known pre-existing psychiatric morbidity or on regular medication for any psychiatric or sexual problems were excluded from the study through skip questions.

The data collection for the study was conducted over a period of 1 month during the gradual easing off of the lockdown restrictions by the Government of India, from July 28, 2020, 12:00 h to August 29, 2020, 23:59 h. The sample size for this study was calculated based on the proportion of sexual dysfunction from a pilot study conducted beforehand. Based on the proportion of 14.40%, with 90% power of the study and 5% precision, with a design effect of 2, the minimum required sample size was noted to be 1,365. From the location data of social media users (Facebook, Twitter, LinkedIn, and Instagram), a list of respondents residing in greater Kolkata region was prepared. From this list, considering 10% non-response, 1,500 participants were shared the questionnaire digitally. Ultimately, a total of 1,376 completed responses were obtained and analyzed.

### Tools and Measurements

The study tool was a pre-designed, pre-tested validated questionnaire with four sections prepared in the English language. The Section Introduction consisted of socio-demographic characteristics like age, gender, education, occupation, and income. It also comprised questions on comorbidities, smoking habits, and practice of COVID-appropriate behaviors. Section Methods assessed mental health status of the participants in terms of depression, anxiety, and stress with the help of the DASS21 questionnaire (26). Section Results was about the questions on sexual health, which was assessed using the Arizona Sexual Experience Scale (ASEX). In Section Discussion, the WHOQOL-BREF scale was used to assess the quality of life (27).

#### Depression Anxiety Stress Scale (DASS)

The 21-item DASS tool (DASS-21) measured depression, anxiety, and stressed with its subscales, each comprising seven items. Response to each item was noted on a 4-point Likert scale from 0 to 3, with a higher score indicating depression or anxiety or stress as per the respective subscale corresponding to the items. In the current sample, Cronbach's alpha values were found to be 0.88 for stress subscale, 0.79 for anxiety, and 0.83 for depression subscales. For each subscale, the respective item-specific scores were summated, and then, resultant scores were doubled. Depression, anxiety, and stress as per this instrument were classified in five resulting categories, i.e., normal (total score: 0–9 for depression, 0–7 for anxiety, and 0–14 for stress subscales), mild (total score: 10–13 for depression, 8–9 for anxiety, and 15–18 for stress subscales), moderate (total score: 14–20 for depression, 10–14 for anxiety, and 19–25 for stress subscales), severe (total score: 21–27 for depression, 15–19 for anxiety, and 26–33 for stress subscales), and extremely severe (total score ≥28 for depression, ≥20 for anxiety, and ≥34 for stress subscales, respectively) (26).

#### Arizona Sexual Experience Scale (ASEX Scale)

ASEX is a five-item self-report inventory, with each item measured on a 6-point Likert scale. Sexual function in men and women were measured regardless of their sexual orientation or relationship with a partner. It measured the quality of sexual functioning in terms of five questions, with each question representing one domain, i.e., sexual drive, arousal, penile erection/vaginal lubrication, ability to reach orgasm, and satisfaction from orgasm. The scores in each of the items were aggregated. Clinical sexual dysfunction was identified if a total score of >19 was observed, and/or in any one item score was >5, and/or in any three items, a score of >4 was noted (28). The Cronbach's alpha value for ASEX in the study sample was 0.83.

#### WHO Quality of Life Questionnaire (WHOQOL-BREF)

The WHOQOL-BREF instrument comprises 26 items, which measure the following broad domains: physical health, psychological health, social relationships, and environment. In each domain, the domain-specific item scores are summed up to denote the domain-specific score. This domain-specific score is a measure of quality of life in that particular domain only. The overall quality of life is measured by summing up all the item-specific scores. The higher the score, the better is the quality of life in any domain or overall (27). The overall Cronbach's alpha value for WHOQOL-BREF was observed to be 0.91, with the alpha value for the subscales ranging between 0.85 and 0.93.

### Statistical Analysis

The statistical analysis was conducted in STATA 14.2 (StataCorp., College Station, TX, USA). Considering the variables of interest, the Generalized Structural Equations Model (GSEM) was utilized to analyze the relationships between the variables. The main GSEM predicted the WHOQOL-BREF total score in terms of sexual dysfunction, depression, anxiety, stress, and other clinical and behavioral variables. In the same model, sexual dysfunction was in turn predicted by depression, anxiety, stress, and selected clinical and behavioral factors. On the other hand, five separate GSEMs were developed to predict each component of sexual dysfunction. In each of these models, the major predictors were depression, anxiety, and stress and also other clinical and behavioral factors. Depression, anxiety, and stress each were predicted by socio-demographic and behavioral factors. The categorical variables with ordinal measurements, e.g., depression, anxiety, stress, loss of income during lockdown (in comparative percentage), and income ranges, were incorporated in the models using probit link. The variables with nominal measurements (e.g., education, marital status, etc.) and the dichotomous variables logit links were used. Age was taken as a continuous variable with identity link. Coefficient (Coef.) with 95% confidence interval (95% CI) has been considered as the measurement of effect. The main effects, i.e., effect of the major predictors on different outcomes (i.e., quality of life, sexual dysfunction and its components), are presented in the results section, along with the reference categories for the predictor variables in these models.

## Results

### Background Characteristics

Table 1 summarizes the socio-demographic background of the participants. Out of the total 1,376 respondents, 80.52% were male, 65.98% were married, 48.54% were graduates, while majority had been working in private companies (42.73%). The mean age of the participants was 34.42 (±9.34) years. Majority (61.63%) of the respondents belonged to the age group of 19–35 years. Among the respondents, 10.76% had lost their jobs during the lockdown. While majority (41.86%) reported that their monthly family income did not decrease during the lockdown, 17.15%, on the other hand, reported that their incomes decreased by more than half of their pre-lockdown income. However, 36.63% of the respondents had a monthly family income of >50,000 Rupees per month.

**Table 1:** Background characteristics of the study participants.

**Background characteristics**	***N***	**%**
**Age** [34.42 (±9.34) years]		
19–35 years	848	61.63
36–50 years	460	33.43
51–65 years	56	4.07
>65 years	12	0.87
**Gender**		
Male	1,108	80.52
Female	268	19.48
**Marital status**		
Unmarried	417	30.31
Married	908	65.99
Separated	28	2.03
Divorced	16	1.16
Widow	7	0.51
**Education**		
Secondary level	16	1.16
Higher-secondary level	84	6.10
Graduation level	680	49.42
Post-graduation level	596	43.31
**Occupation**		
Laborer	24	1.74
Businessman	132	9.59
Government sector employees	256	18.60
Private sector employees	588	42.73
Professionals	68	4.94
Other	308	22.38
**Monthly family income (in Rupees)**		
≤ 3,000	84	6.10
3,001–10,000	140	10.17
10,001–25,000	272	19.77
25,001–50,000	376	27.33
>50,000	504	36.63
**Loss of monthly income during lockdown**		
No change	576	41.86
≤ 10%	168	12.21
11–25%	212	15.41
26–50%	184	13.37
>50%	236	17.15

*N, number of respondents; %, percentage of respondents in each category*.

### Clinical and Behavioral Profile of the Participants

Table 2 shows the clinical and behavioral characteristics of the study participants. Majority (79.02%) did not report any comorbidities. However, some had diabetes (10.47%), some had pulmonary comorbidities (9.59%), and few of the respondents had diagnosed chronic kidney disease (1.74%). Majority of the respondents did not have depression (59.30%), anxiety (52.33%), or stress (44.48%). Mild and moderate levels were the most common findings among those who had depression, anxiety, or stress. Regarding maintaining COVID-19 appropriate behaviors, most of the participants were observing the recommended norms.

**Table 2:** Clinical and behavioral profile of the respondents.

**Clinical and behavioral factors**	***N***	**%**
Known comorbidities*		
Diabetes	144	10.47
Asthma/COPD	132	9.59
Chronic kidney disease	24	1.74
**Smoking history**		
Presently a smoker	616	44.77
Not a smoker	760	55.23
COVID-19 preventive behaviors*		
Regular use of masks	1,352	98.30
Maintaining physical distancing	1,103	80.20
Regular hand wash by soap and water and/or use of sanitizer	1,252	91.00
Maintaining cough hygiene	1,348	98.00
Regular examination with thermal scanner	364	26.50
**Depression**		
Normal	816	59.30
Mild	192	13.95
Moderate	188	13.66
Severe	88	6.40
Extremely severe	92	6.69
**Anxiety**		
Normal	720	52.33
Mild	184	13.37
Moderate	308	22.38
Severe	76	5.52
Extremely severe	88	6.40
**Stress**		
Normal	612	44.48
Mild	488	35.47
Moderate	140	10.17
Severe	100	7.27
Extremely severe	36	2.62

*N, number of respondents; %, percentage of respondents in each category; COPD, chronic obstructive pulmonary disease*.

* *Multiple response*.

### Sexual Dysfunction and Its Associated Factors

**Figure 1:**
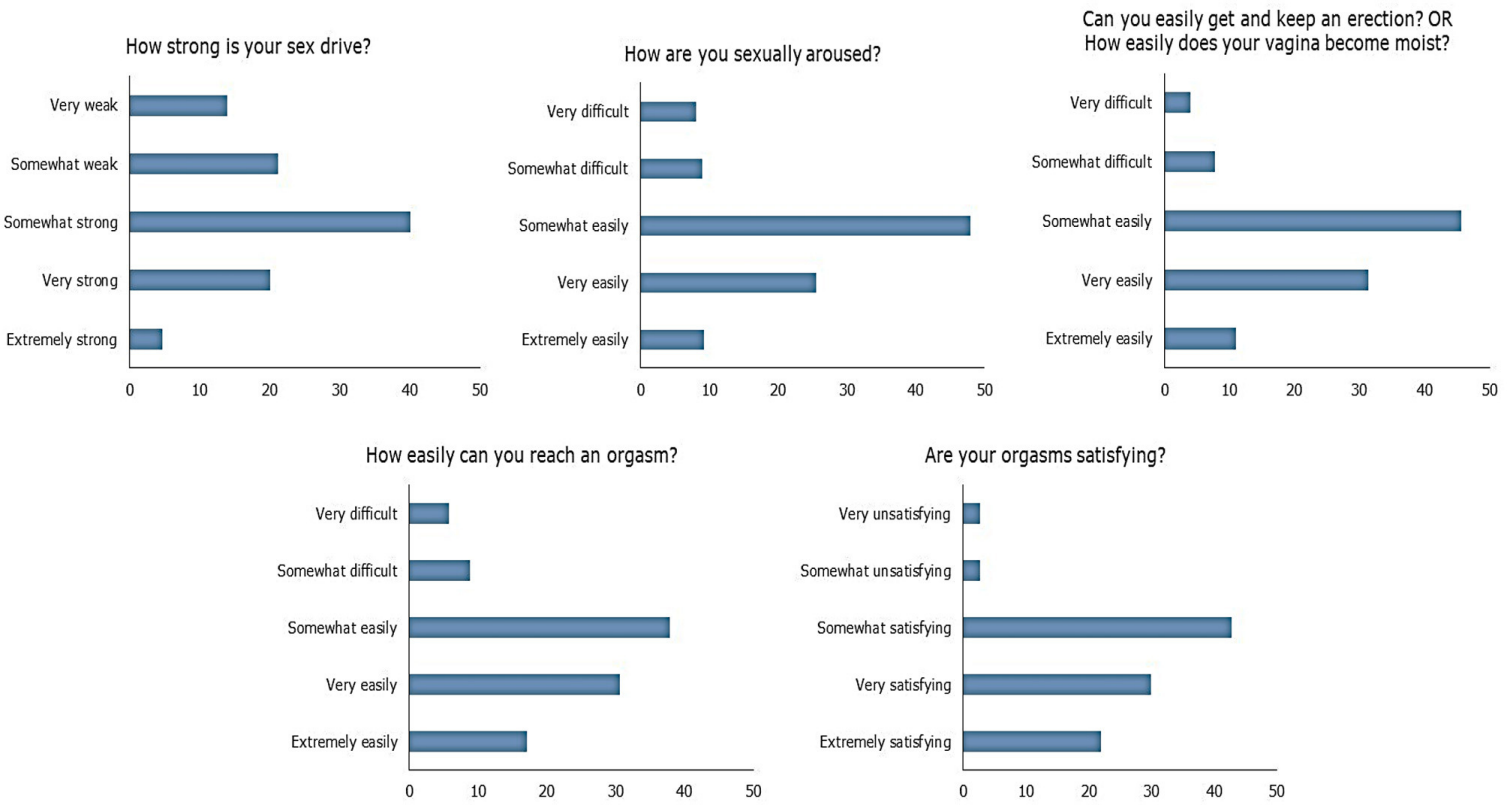
Summary of responses to the items in Arizona Sexual Experience Scale (ASEX).

Among the respondents, 27.18% had sexual dysfunction as per the ASEX instrument. The item-wise responses of ASEX are depicted in Figure 1. Table 3 summarizes the key associations in terms of the components of ASEX and also overall sexual dysfunction from five separate GSEMs and the main GSEM, respectively. The relationships of socio-demographic and clinical factors with the components of ASEX and also with overall presence of sexual dysfunction are depicted. Increase in age and female gender were associated with sexual dysfunction overall, and also with all its components independently. The presence of depression adversely affected ease of achieving orgasm and satisfaction from orgasm. Depression was overall statistically associated with sexual dysfunction among the respondents. In addition, the presence of any comorbidity was associated with sexual dysfunction.

**Table 3:** Generalized structural equations model analysis showing relationship of socio-demographic and clinical factors with sexual dysfunction among the respondents.

	**Desire**		**Excitement**		**Erection/lubrication**		**Orgasm**		**Orgasmic satisfaction**		**Overall sexual dysfunction**	
	**Coef. (95% CI)**	***p*** **-value**	**Coef. (95% CI)**	***p*** **-value**	**Coef. (95% CI)**	***p*** **-value**	**Coef. (95% CI)**	***p*** **-value**	**Coef. (95% CI)**	***p*** **-value**	**Coef. (95% CI)**	***p*** **-value**
Depression^a^	0.09 (−0.03, 0.22)	0.138	0.13 (−0.01, 0.26)	0.075	**0.19 (0.06, 0.32)**	**0.003**	**0.45 (0.19, 0.71)**	**0.001**	**0.58 (0.35, 0.81)**	**0.000**	**0.30 (0.14, 0.46)**	**0.000**
Anxiety^a^	−0.10 (0.23, 0.03)	0.146	−0.10 (−0.22, 0.02)	0.100	−0.10 (−0.21, 0.01)	0.071	0.04 (−0.17, 0.26)	0.689	**0.28 (0.09, 0.47)**	**0.005**	0.02 (−0.14, 0.17)	0.834
Stress^a^	0.13 (−0.03, 0.29)	0.113	0.07 (−0.10, 0.24)	0.399	0.05 (−0.12, 0.22)	0.549	−0.05 (−0.31, 0.22)	0.727	0.07 (−0.21, 0.34)	0.636	−0.03 (−0.24, 0.18)	0.783
Age^b^	**0.05 (0.04, 0.06)**	**0.000**	**0.04 (0.02, 0.05)**	**0.000**	**0.04 (0.03, 0.06)**	**0.000**	**0.04 (0.02, 0.06)**	**0.001**	**0.04 (0.02, 0.06)**	**0.000**	**0.05 (0.03, 0.06)**	**0.000**
Sex^c^	**0.42 (0.13, 0.71)**	**0.004**	**0.94 (0.61, 1.26)**	**0.000**	**1.33 (1.03, 1.63)**	**0.000**	**2.07 (1.64, 2.50)**	**0.000**	**0.83 (0.36, 1.31)**	**0.001**	**0.87 (0.51, 1.23)**	**0.000**
Marital status^d^	–**0.55 (**–**0.79**, –**0.32)**	**0.000**	–**0.58 (**–**0.84**, –**0.32)**	**0.000**	–**0.39 (**–**0.67**, –**0.12)**	**0.005**	−0.19 (−0.72, 0.33)	0.472	0.00 (−0.58, 0.58)	0.996	−0.28 (−0.61, 0.05)	0.096
Presence of comorbidities^c^	0.16 (−0.11, 0.43)	0.244	0.25 (−0.05, 0.55)	0.103	**0.53 (0.22, 0.84)**	**0.001**	0.24 (−0.12, 0.59)	0.188	0.32 (−0.08, 0.71)	0.122	**0.43 (0.13, 0.73)**	**0.005**
Smoking status^c^	0.19 (−0.03, 0.40)	0.094	−0.05 (−0.28, 0.18)	0.679	−0.24 (−0.47, −0.01)	0.040	−0.17 (−0.48, 0.13)	0.267	−0.25 (−0.56, 0.06)	0.120	−0.22 (−0.50, 0.06)	0.120

*Coef., coefficient of regression equations; CI, confidence interval*.

a *Probit linkage used for ordinal level of measurement for these variables with assumption of homogeneity of risk. The reference categories for Depression, Anxiety, and Stress are the “Normal” groups*.

b *Age was taken in a continuous scale of measurement assuming Gaussian distribution and using identity linkage*.

c *These variables were measured dichotomously. For sex, “male” was the reference category, while in the case of presence of comorbidities, and smoking status the reference categories were “absence of any comorbidity” and “non-smoker”, respectively*.

d *For marital status, Divorced, Separated, and Widow categories were merged into “previously married” category yielding three nominal level categories, viz., unmarried, currently married, and previously married. For this variable, also uniformity of risk was assumed, and the coefficient was calculated taking the “Unmarried” group as reference*.

*The bold values indicate statistically significant*.

### Association of Sexual Dysfunction With Quality of Life of the Respondents

Regarding quality of life, the respondents had a mean score of 73.57 (±13.50). Table 4 depicts the results of GSEM analysis showing association between the quality-of-life score with sexual dysfunction, adjusted for other relevant variables. Sexual dysfunction was not observed to be statistically associated with quality of life among the respondents. However, depression and stress with the exception of anxiety emerged as statistically significant factors for poor quality of life among the respondents. Maintaining a COVID-19 preventive behavior was also not statistically associated with quality-of-life score.

**Table 4:** Relationship between socio-clinical factors and quality-of-life of the respondents as per generalized structural equations model analysis.

**Variables**	**Coef. (95% CI) for a higher QOL score**	***p*** **-value**
Sexual dysfunction^a^	0.05 (−1.24, 1.35)	0.935
Depression^b^	–**3.92 (**–**4.61**, –**3.24)**	**0.000**
Anxiety^b^	0.18 (−0.51, 0.88)	0.606
Stress^b^	–**3.01 (**–**3.99**, –**2.04)**	**0.000**
Age^c^	0.03 (−0.06, 0.11)	0.550
Sex^a^	1.20 (−0.37, 2.78)	0.135
Marital status^d^	1.12 (−0.32, 2.56)	0.127
Income loss^b^	–**0.93 (**–**1.36**, –**0.50)**	**0.000**
Lost job^a^	**5.71 (3.72, 7.69)**	**0.000**
Maintaining COVID protocol^a^	−1.10 (−2.69, 0.49)	0.174
Presence of any comorbidity^a^	–**2.81 (**–**4.36**, –**1.27)**	**0.000**
Monthly family income^b^	0.43 (−0.09, 0.94)	0.107
Smoking status^a^	1.10 (−0.10, 2.29)	0.071
Educational level^e^	−0.66 (−1.55, 0.24)	0.150

*Coef., coefficient of regression equations; CI, confidence interval; QOL, quality of life*.

a *These variables were measured dichotomously. For sex, “male” was the reference category, while in the case of lost job, presence of comorbidities, and smoking status, the reference categories were “did not lose job,” “absence of any comorbidity,” and “non-smoker,” respectively. “No sexual dysfunction' was the reference category for sexual dysfunction*.

b *Probit linkage used for ordinal level of measurement for these variables with assumption of homogeneity of risk. The reference categories for Depression, Anxiety, Stress are the “Normal” groups. For Monthly family income and Income loss, the reference categories were, “ ≤ 3,000 Rupees per month” and “No Change,” respectively*.

c *Age was taken in a continuous scale of measurement assuming Gaussian distribution and using identity linkage*.

d *For marital status, Divorced, Separated, and Widow categories were merged into “previously married” category yielding three nominal level categories, viz., unmarried, currently married, and previously married. For this variable, also uniformity of risk was assumed, and the coefficient was calculated taking “Unmarried” group as reference*.

e *Secondary and higher-secondary categories were combined into “below graduation” category, which was the reference category*.

*The bold values indicate statistically significant*.

## Discussion

### Findings in Light of Existing Literature

#### Comorbidities in the Case of Sexual Dysfunction

In a sample of 1,376 individuals residing in Kolkata, 27% had SD as per ASEX scoring criteria. A pre-COVID-19 era epidemiological study conducted in a South Indian population using the same scale (ASEX) reported its prevalence to be 21% (male) and 15% (female) (29). The psychometric construct of ASEX typically considers high scores to demarcate SD. However, it was proposed by the makers of ASEX to consider subjects having premature ejaculation or spontaneous orgasm (reflected in extremely low ASEX scores) to have sexual dysfunction, given that sexual dysfunction can involve both hyperfunction and hypofunction (28). Older men, diabetic male patients, men with cardiovascular disease, men who are overweight/obesity, and those with multiple comorbidities are at highest risk for suffering serious complications secondary to COVID-19, and they are also at risk for erectile dysfunction (ED), which is the most common sexual health concern among men (30). ED is an excellent surrogate marker of systemic health, in general, and vascular performance, in particular (endothelial dysfunction resulting in erectile dysfunction and vice versa) (31).

Approximately 20% of the respondents suffered from chronic health issues: diabetes (10.5%), COPD and asthma (9.6%), and hypertension, which exemplifies the emergence of non-communicable diseases, especially diabetes, in recent times. This finding in a population who are predominantly young is a grim reminder of the warning that India is the new diabetes capital of world (32). The finding that almost all are following COVID protocol is encouraging, yet subjected to possible conformity and response bias as observed by prior epidemiological studies (33). No statistical relation was found between sexual dysfunction and maintaining COVID protocol: sexual abstinence owing to fear of infection and non-compliance to precautionary measure resulting from sexual frustration are enlisted by Banerjee in the “Probable sexuality and intimacy-related issues during pandemic” (34). There are not enough data to comment on the first concern, but the presence of the latter issue is not established with the study population.

#### The Role of Depression, Anxiety, and Stress

The prevalence of some level of depression was ~40% in the study population (12% were suffering from severe and very severe depression), whereas almost half of the population (47%) were experiencing some level of anxiety. This finding is similar to the earlier works carried out in India (35, 36). Recent meta-analysis found that one-third of the population are suffering from depression and anxiety (37). Among the population, this result, which surfaced in a screening survey, warrant us about the treatment gap in mental illness in India, which is as high as 80%, and this is bound to increase further in post-COVID scenario (38).

The current study found that people suffering from depression have more chance of having sexual dysfunction. The findings are in consonance with the current literature. While a Polish study established the correlation of depression and SD in women, in the study by Fang et al., this association was established among the men (15, 17). Both behavioral and biological models have been proposed to explain this relationship. The behavioral model postulates that patients with depression tend to engage in negative thought and are less confident, which results in performance anxiety that further reduces erectile function, whereas the biological model postulates that depression affects the hypothalamic–pituitary–adrenocortical (HPA) axis, leading to excess catecholamine production, which in turn leads to poor cavernosal muscle relaxation and ED.

Anxiety and stress were not found to be significantly associated with SD in the present study. Culha et al. in this regard found out that during the pandemic, anxiety was associated with SD but not depression or stress (13). On the other hand, in a study from China, anxiety was correlated with SD in men (15). It is easier to blame COVID-related stress for poor sexual health, but the relationship between the two is complex. Increased levels of stress can reduce sexual urge, but social distancing and stressful circumstances can also increase the need for emotional bonding. Online communication as a form of prosocial behavior at the time of a stressful event can encourage and strengthen people's bonds. Regardless of one's relationship status, expressing intimacy is vital to sustaining positive coping and psychological wellbeing. It is interesting to observe a paradoxical increase in pornography consumption globally during peak of pandemic. As reported, worldwide traffic to pornographic websites skyrocketed compared to the situation before the pandemic during the month of February and March 2020 when Europe was under full lockdown (39). The “Dual Control Model of Sexual Response” theory might be a plausible explanation to the contradictory and paradoxical relationship between stress and sexuality. From this lens, people who tend to be sexually inhibited would have a more difficult time becoming aroused in stressful situations, whereas times of anxiety and stress may amplify sexual arousal in people who are usually easily excitable (30).

#### Association of Age and Gender

Female gender appeared as a significant correlate of SD in the present study. This supports the finding by other researchers that sexual problems are much under-reported among women (40). Desire, arousal, erection, and orgasm difficulties, all these are significantly predicted by female gender and being ever married. Among other authors who studied sexual behavior during COVID pandemic, few studies indicated reduction in sexual function in women and an increase in female sexual dysfunction in general (41, 42). Other researchers, in contrast, noted that during the pandemic, despite a sharp decrease in the quality of sexual life in women, sexual desire and frequency of intercourse significantly increased (43). However, in a study during the pandemic in Turkey among healthcare workers, male gender was identified as a significant factor for sexual dysfunction (13). This contrast may again be attributed to a probable under-reporting of SH status of women.

A common disorder in the spectrum of female sexual dysfunction (FSD) is decreased vaginal lubrication (which is deemed equivalent to erection phase in men) during the COVID-19 pandemic, which in turn leads to other problems, such as dyspareunia, orgasm dysfunction, vagina irritation, or increased risk of vaginitis (44). From a traditional outlook, “lockdown” for a man provided longer time to interact with the spouse, but for most women, it meant an increase in the workload in all spheres (domestic, working from home professionally, and looking after children attending online classes). Ironically, when one partner is looking to have more sex and the other is distracted, preoccupied, or otherwise disengaged, the issue of sexual desire discrepancies arises (18). Additionally, the presence of everyone at home throughout the day combined with higher work pressure led to frequent interpersonal issues and more chances of domestic violence (45). Interestingly, the smallest decline in sexual activity was noted in women who were working outside the home (44). Pre-pandemic research involving ASEX to identify sexual dysfunction in patients of the schizophrenia spectrum found a much higher rate in women (79.2%) than in men (33.3%). Indeed, women reported high frequencies of sexual dysfunctions in all stages of sexual activities (sexual drive, arousal, vaginal lubrification, ability to reach orgasm, and satisfaction with orgasm) (46). This difference between genders can be attributed to biopsychosocial factors: sexual hormones (estrogens vs. androgens), sexual education (repressing vs. permissive), and environment (controlling vs. stimulant) (47).

Increasing age emerged as a significant predictor of difficulties in all spheres of sexual cycle (desire, arousal, erection, and orgasm). To elaborate the association of increasing age with SD, prior authors mentioned that sexual dysfunction worsens with age. Thirty-nine percent of 40-year-olds have some degree of erectile dysfunction (5% are completely impotent), but by the age of 70, two-thirds have some degree of erectile dysfunction and complete impotence triples to 15% (48). Occurrence of SD with increasing age might be explained by poorer vascular health and endothelial dysfunction as explained earlier.

#### The Issue of Quality of Life

In our study, SD was not statistically associated with quality of life (QoL). The association of QoL with ED studied earlier mostly showed deleterious effect of ED on life satisfaction and overall QoL. Rosen, on the other hand, suggested an indirect pathway model that hypothesized that changes in erectile function (EF) through treatment were associated with improved mood and quality of sexual life, which resulted in improved partner satisfaction, family life, and overall life satisfaction. These data suggest that QoL changes associated with ED therapy may be mediated by changes in sexual function, mood, and family relationships (21). UK-based survey demonstrated that men with ED and multiple risk factors had poorer QoL than men with ED and no risk factors (49).

Lastly, summarizing the somewhat contradictory findings of studies done by authors during current pandemic: the opportunity for physical intimacy that prolonged confinement and forced coexistence resulting from lockdown has created is undeniable. An online survey from the United Kingdom showed that, during the period of self-isolation, about two-fifth of the respondents reported to have had sex at least once in a week (50). But for others, stress and extended proximity to one's partner exacerbate differences in desire, as cited by a recent study conducted in India exploring the same circumstance (51). Yet, it led to an improvement in overall relationship with the partners, communication with the partner, and reduction in the interpersonal conflicts.

### Strengths and Limitations

This is not the first study to investigate sexual activity during the COVID-19 self-isolation/social distancing, but research data in the subcontinent in this topic are not vast. The findings, despite the novelty, must be interpreted in light of the limitations. Since this was an online survey, youths in their 30s constituted majority of the participants considering their familiarity with social media. In addition, only a population fluent in English participated. Taboo and stigma attached to sexuality might have been the reason that limited female volunteers more in the survey, given that 80% of the participants were male. This lack of female participation has been observed by prior researchers working on sexual side effects of antipsychotics (52). There is scope of measurement bias, as participants were asked to self-report their sexual activity, mental health status, and quality of life, all of which may be subjected variable mental computing mechanisms at the time of reporting, resulting in a systematic deviation. While use of GSEM makes the analysis robust, introduction of different levels of variables may have affected the model estimation, which was outside the scope of this study to analyze further. The analyses were cross-sectional, and thus, it is not possible to determine trajectories of sexual activity during the current pandemic. Pre-COVID data were not collected for comparison, and significant areas of sexual behavior queries like masturbatory habit, consumption of pornographic material, or homosexual relationship were not included in the questionnaire.

### Implications/Recommendations

Findings from the present study shed light on sexual activity during COVID-19 self-isolation/social distancing among Indian subpopulation. These findings suggest that interventions to promote good mental and physical health during the COVID-19 self-isolation/social distancing period should take into account positive sexual health. The psychological burden self-isolation/social distancing must not be translated into the vicious cycle of poor state of sexual health to poorer state of mental health. A view of sex and intimacy as a mode of positive coping and social connection building might be promoted under a non-prejudiced mindset. Addressing the unmet need of women suffering from sexual disorder is the need of the hour. One must be vigilant to the burden of non-communicable disease and of mental morbidity in youth that emerged as a collateral finding.

## Data Availability Statement

The original contributions presented in the study are included in the article/supplementary material, further inquiries can be directed to the corresponding author/s.

## Ethics Statement

The studies involving human participants were reviewed and approved by Institutional Ethics Committee, Diamond Harbour Government Medical College and Hospital. The patients/participants provided their written informed consent to participate in this study.

## Author Contributions

SC and RB: conceptualization, methodology, investigation, writing—original draft, and project administration. AC: methodology, validation, and writing—review and editing. AL: conceptualization, methodology, formal analysis, writing—review and editing, and supervision. AD: software, formal analysis, and writing—review and editing. All authors contributed to the article and approved the submitted version.

## Conflict of Interest

The authors declare that the research was conducted in the absence of any commercial or financial relationships that could be construed as a potential conflict of interest.

## Publisher's Note

All claims expressed in this article are solely those of the authors and do not necessarily represent those of their affiliated organizations, or those of the publisher, the editors and the reviewers. Any product that may be evaluated in this article, or claim that may be made by its manufacturer, is not guaranteed or endorsed by the publisher.
